# The proline-rich antimicrobial peptide B7-005: low bacterial resistance, safe for human cells and effective in zebrafish embryo bacteraemia model

**DOI:** 10.1098/rsob.240286

**Published:** 2024-12-04

**Authors:** Adriana Di Stasi, Sara Bozzer, Sabrina Pacor, Luigi de Pascale, Martino Morici, Lara Favero, Mariagiulia Spazzapan, Silvia Pegoraro, Roberta Bulla, Daniel N. Wilson, Paolo Macor, Marco Scocchi, Mario Mardirossian

**Affiliations:** ^1^ Department of Life Sciences, University of Trieste, 34127 Trieste, Italy; ^2^ Institute for Biochemistry and Molecular Biology, University of Hamburg, 20146 Hamburg, Germany; ^3^ Department of Medicine, University of Padova, 35122 Padova, Italy; ^4^ Institute for Maternal and Child Health Irccs Burlo Garofolo, 34137 Trieste, Italy

**Keywords:** antimicrobial peptide, proline-rich, antibiotic resistance, protein synthesis, drug discovery

## Abstract

Proline-rich antimicrobial peptides (PrAMPs) have gained attention due to their antimicrobial properties and low cytotoxicity. B7-005, a small optimized PrAMP, exhibits a broader spectrum of activity than native PrAMPs, due to an antimicrobial mechanism based on inhibiting prokaryotic protein synthesis and destabilizing bacterial membranes. However, the toxicity and the *in vivo* efficacy of B7-005 remain poorly understood, so *in vitro* and *in vivo* microbiology and toxicology experiments were used to assess its suitability as an anti-infective agent. The incidence of resistance towards B7-005 by *E. coli* was lower than for other PrAMPs and antibiotics; moreover, it maintained antimicrobial activity in the presence of human serum. B7-005 exerted its antimicrobial effect at a much lower concentration than those causing harmful effects on four different cell types, such as membrane permeabilization or non-lytic depolarization of mitochondria. The latter effect may be related to the inhibition of eukaryotic protein synthesis by B7-005 observed *in vitro*. In a zebrafish embryo model, B7-005 was well tolerated and reduced mortality from pre-existing *E. coli* bacteraemia. Overall, B7-005 was safe for human cells and effective against systemic infection *in vivo*, making it a promising lead for developing new antibiotics.

## 1. Introduction

Persistent use of antibiotics has led to the emergence of multidrug-resistant (MDR) and extensively drug-resistant (XDR) bacteria that render even the most effective drugs ineffective [1–3]. Among the alternatives under study for the development of new antimicrobial therapeutics, antimicrobial peptides (AMPs) have gained increased attention as innovative drug candidates for the treatment of infectious diseases [4,5]. AMPs are small host defence peptides with potent antibacterial, antiviral and antifungal activity, that are ubiquitous in multicellular eukaryotes and are expressed to combat infections [6]. Although membrane targeting and permeabilization were considered the only mechanism of action for AMPs [7], in the past few years, there has been increasing evidence that AMPs also have other modes of action [7–9]. In particular, proline-rich AMPs (PrAMPs) have been shown to pass through the membrane and inhibit protein synthesis by binding to the ribosome [10–15]. Efficient internalization of PrAMPs generally requires the membrane transporter SbmA, which is present in some Gram-negative bacteria, such as *E. coli* [16]. As a consequence, PrAMPs exhibit a narrower spectrum of activity than most membrane-directed AMPs. While the specificity of PrAMPs reduces the number of sensitive target pathogens, it also has a benefit by significantly reducing the risk of adverse effects of PrAMPs towards eukaryotic cells—a common problem of membrane-targeting AMPs [17,18]. Nevertheless, some PrAMPs still exert membranolytic activity, especially at higher concentrations, which can lead to some cytotoxicity against eukaryotic cells. This has been addressed by generating shorter PrAMPs that maintain antimicrobial activity but are better tolerated by human and other eukaryotic cells [14], thus making short ribosome-targeting PrAMPs a very attractive lead for the development of new and improved antimicrobial drugs.

In a previous work, we systematically modified Bac7(1-16), an active N-terminal fragment of the natural PrAMP full-length Bac7 [19], to identify the amino acid residues essential for inhibiting protein synthesis, with the goal to discover new derivatives with improved antimicrobial activity [20]. Among the identified derivatives, B7-005 was the most interesting because it maintained its inhibitory activity against protein synthesis but also exhibited a moderate membrane-permeabilization effect that conferred antimicrobial activity of B7-005 even against strains lacking SbmA [20]. B7-005 (**W**RIR**R**R**W**PRLPRPR**W**R) contains four substitutions relative to the parent peptide Bac7(1-16) (RRIRPRPPRLPRPRPR). The introduction of Arg and Trp residues at specific positions in Bac7(1-16) conferred antimicrobial activity against a broader range of bacterial pathogens than native Bac7(1-16). Preliminary tests on a human cell line suggested that the enhanced antibacterial activity of B7-005 did not increase cytotoxicity relative to Bac7(1-16) [20]. For these reasons, the biological properties of B7-005 need to be characterized in greater detail using a larger panel of eukaryotic cells. There is also minimal information regarding the basic pharmacodynamic parameters of B7-005, which may deeply influence and fine-tune the activity of the peptide drug, for example, potential interaction with serum proteins [21]. Finally, B7-005 should also be tested within a whole organism to verify its potential as a novel antimicrobial agent.

In this study, B7-005 was evaluated for its antibacterial activity in human serum and for its tendency to select *E. coli*-resistant mutants. In addition, the potentially adverse effects of B7-005 were analysed using four different human cell types. A long 24 h exposure and a short 1 h exposure to highly concentrated peptide suggested that B7-005 was well tolerated by human cells. The cytotoxic mode of action of B7-005 was investigated using a very high amount of peptide that exerted a variable cell-dependent toxic effect, mainly targeting the plasma membrane but that may involve also mitochondria. Furthermore, B7-005 PrAMP was effective *in vivo*, improving the survival of zebrafish embryos in a model of *E. coli* bacteraemia. The desirable properties showed by B7-005 provide a solid basis for its further development as a new antibacterial agent.

## 2. Material and methods

### 2.1. Peptides and antibiotics

All solid-phase synthesized unlabelled peptides (B7-005, Bac7(1-16) and Bac7(1-35)) with a purity of >95% were purchased from NovoPro Bioscience (Shanghai, China). B7-005-BY was purchased from JPT (Berlin, Germany), synthesized on solid-phase, purified to >95% and labelled with BodipyFL, linking the fluorophore to an additional C-terminal cysteine added ad hoc. All the peptides were resuspended in 500 µl of 10 mM HCl and lyophilized. Resuspension and lyophilization were repeated three times to remove TFA. Peptides were then resuspended in sterile Milli-Q water and quantified by their absorbance at 214 and 280 nm. The molar extinction coefficient was calculated in accordance with Kuipers & Gruppen [22]. Chloramphenicol was dissolved in ethanol (95% v/v) at a concentration of 3.5 mg ml^−1^. Rifampicin was solved in dimethyl sulfoxide (DMSO, Sigma) at a concentration of 10 mg ml^−1^. For both antibiotics, the less concentrated stocks were diluted in Milli-Q water. Colistin was dissolved in sterile Milli-Q water at 2.5 mg ml^−1^. All antibiotics used were purchased from Sigma-Aldrich (USA).

### 2.2. Bacterial cultures

*Escherichia coli* ATCC 25922 was purchased from the American Type Culture Collection (ATCC) (Manassas, Virginia, USA) and stored at −80°C as glycerol stock until use. Bacterial cultures were grown overnight in sterile Müller–Hinton broth (MHB) (Difco Inc.) at 37°C under agitation (140 r.p.m.). The next day, the cultures were diluted 1 : 30 in new MHB and incubated as described above for about 2 h (mean logarithmic phase) until they reached an optical density at 600 nm of about 0.3 and then diluted in fresh MHB to the desired concentrations.

### 2.3. Cell lines and growth condition

Chronic lymphocytic leukaemia-derived MEC-1 cells were cultured in suspension using complete RPMI (EuroClone), supplemented with 100 U ml^−1^ penicillin (Sigma), 100 μg ml^−1^ streptomycin (Sigma), 2 mM l-glutamine and 10% (v/v) foetal bovine serum (FBS, EuroClone). A549 human lung carcinoma epithelial cells and immortalized human keratinocytes (HaCaT) were grown in adhesion in complete high glucose Dulbecco’s MEM (DMEM, EuroClone) supplemented with 100 U ml^−1^ penicillin (Sigma), 100 mg ml^−1^ streptomycin (Sigma), 2 mM l-glutamine and 10% (v/v) of FBS. Human umbilical vein endothelial cells (HUVECs) were cultured in Human Endothelial Serum-Free Medium (HESFM, Life Technologies, Carlsbad, CA, USA) supplemented with 20 ng ml^−1^ of epidermal growth factor (EGF), 10 ng ml^−1^ basic fibroblast growth factor (bFGF) (Immunological Sciences), 100 U ml^−1^ penicillin and 100 μg ml^−1^ streptomycin (Sigma-Aldrich) and 10% (v/v) FBS (Life Technologies). All cell lines were grown at 37°C and in the presence of 5% CO_2_. A549 and HaCaT cells were removed from the flask before reaching confluence (~80%) by a 5 min incubation at 37°C with 2 ml of 1× trypsin/EDTA (500 mg l^−1^ trypsin, 371 mg l^−1^ EDTA, EuroClone SpA, Italy), whereas HUVECs were grown until confluence and then detached from the flask by a 2 min incubation at 37°C with 2 ml of trypsin/EDTA (see above) diluted 1 : 2 in sterile PBS. All cell lines were counted in a Bürker chamber and diluted to the desired working concentrations.

MEC-1 and HaCaT cell lines were purchased from the ATCC (Manassas, VA, USA). A549 cell line was purchased from the Deutsche Krebsforschungszentrum (DKFZ) (Heidelberg, Germany). HUVECs were purchased from CLS Cytion Cell Lines Service GmbH (Eppelheim, Germany).

### 2.4. Effects of human serum on the antimicrobial activity of B7-005

The effects of human serum on the antimicrobial activity of B7-005 were assessed by performing a minimum inhibitory concentration (MIC) assay [20] with some modifications. Non-heat-inactivated human serum (Merk Life Science S.r.l., USA) was diluted to 50%, 40%, 30%, 25%, and 20% (v/v) in MHB and the solutions were aliquoted into the wells of a round-bottomed 96-well microtitre plate (Sarstedt, Milan, Italy). Samples with no serum were used for comparison. B7-005 was diluted in MHB added with the proper amount of serum to a concentration of 128 μM, added to the first column of the plate and serially diluted twofold in MHB, added with the proper amount of serum, in the subsequent wells in a final volume of 50 μl. Then 50 μl of a mid-log suspension of 5 × 10^5^ CFU ml^−1^ *E. coli* ATCC 25922 cells prepared in MHB added with the proper amount of serum was added to each well of the plate, halving the final concentration of bacteria and peptide. The plate was incubated at 37°C for 18 h. The MIC value was calculated as the lowest concentration of the compound resulting in complete inhibition of visible bacterial growth. MIC values were determined by performing at least three independent experiments (*n* ≥ 3).

### 2.5. Resistance selection by serial passages in *E. coli* ATCC 25922

The resistance selection assay was performed by modifying a previously described method [23,24]. First, the MIC values of each antimicrobial compound (PrAMP or antibiotic) against *E. coli* ATCC 25922 were determined and recorded as described above. Serial sub-culturing was started by harvesting bacterial cells growing at one-half MIC and inoculating them into fresh MHB. To maintain selective pressure on bacteria, one-half MIC concentration of the test compound was also added to the growth medium. This inoculum was incubated o/n and subjected to another MIC test. After 18 h of incubation, the cells growing at one-half MIC were harvested and used for another similar MIC assay. The process was repeated for 7 or 14 daily passages. The concentrations of the antimicrobial compounds were adjusted during the process to compensate for the increase in MIC values.

### 2.6. *In vitro* cytotoxicity against human cell lines

Cell viability was evaluated by tetrazolium salt (MTT, or MTS for HUVECs only) tests. 100 μl of A549 or HaCaT cells (2 × 10^5^ cells ml^−1^) in complete DMEM were seeded in 96-well flat-bottom microtitre plates (EuroClone SpA, Italy). Plates were incubated o/n at 37°C and 5% CO_2_. The next day, the exhausted medium was removed from each well and replaced with 100 μl of B7-005 prepared at the desired concentration in DMEM. Alternatively, 50 μl of the 2 × 10^6^ cells ml^−1^ MEC-1 cell suspension was aliquoted into the wells of a 96-well flat-bottomed plate (EuroClone SpA, Italy) in RPMI medium. Fifty microlitres of B7-005 in RPMI (concentrated 2× with respect to the desired final concentrations) were then added to the wells. After 20 h of incubation at 37°C and 5% CO_2_, 25 μl of MTT (Merck Life Science S.r.l.) (5 mg ml^−1^ in PBS) were added to each well and the plate incubated for 4 h at 37°C and 5% CO_2_ in the dark. For A549 and HaCaT cells (and not for MEC-1), the solution was carefully removed from each well to prevent loss of MTT crystals, and each well was then washed with 100 μl sterile PBS. Then, for all cell lines, 100 μl IGEPAL (Merk Life Science S.r.l.) (10% w/v in 10 mM HCl) was added to each well to dissolve the MTT crystals and incubated o/n at 37°C and 5% CO_2_. The next day, absorbance was measured at 570 nm using a Nanoquant Infinite-M200Pro plate reader (Tecan, Switzerland).

HUVECs, 7500 cells per well, were seeded in HESFM with 10% FBS in a 96-well flat-bottomed microtitre plate (EuroClone SpA, Italy). The plate was incubated o/n at 37°C and 5% CO_2_. The next day, the exhausted medium was replaced with 100 μl of B7-005 prepared at the desired concentration in HESFM supplemented with 10% FBS. After 24 h, the medium containing the peptide was removed and 200 μl per well of a solution of MTS (Promega Corporation, Madison, USA), diluted 1 : 5 in PBS containing 4.5 g l^−1^ glucose, 0.7 mM Ca^2+^ and 0.7 mM Mg^2+^, was added. The plate was then incubated for 2 h at 37°C in a humidified 5% CO_2_ atmosphere, and the absorbance was measured at 490 nm, using a spectrophotometer (PowerWaveX, Bio-Tek Instruments). The cytotoxicity was calculated by comparing the absorbance of peptide-treated samples with that of the untreated control and expressed in percentage of vitality. The results are the average ± s.e.m. of at least three independent experiments conducted in triplicate.

### 2.7. Eukaryotic cell membrane integrity

Membrane integrity of the eukaryotic cells was evaluated by flow cytometric assays measuring propidium iodide (PI) uptake. 600 μl of a suspension of 1.2 × 10^5^ cells ml^−1^ (A549, HaCaT and HUVECs) were seeded in a growth medium per well in a 24-well flat-bottom microtitre plate (EuroClone SpA, Italy) and the plate incubated o/n at 37°C and 5% CO_2_. The following day, the medium was removed, replaced with an equal volume of DMEM containing B7-005 at selected concentrations and the plate was incubated for 24 h at 37°C and 5% CO_2_. 5 × 10^5^ MEC-1 cells were transferred in a 24-well flat-bottom microtitre plate (EuroClone SpA, Italy) at a volume of 250 μl per well in RPMI. A serial twofold dilution of B7-005 was prepared and aliquots of 250 μl were transferred into each cell-containing well, and the plate was incubated for 24 h at 37°C and 5% CO_2_. At the end of the exposure to B7-005, A549 and HaCaT cells were detached with 250 μl of 1× trypsin/EDTA (see §2.3) and incubated for 5 min at 37°C, whereas HUVECs were incubated for 1 min at 37°C with 250 μl of trypsin/EDTA (see above) diluted 1 : 2 in PBS. Then, detached cells were immediately transferred into a sterile tube, added with PI to a final concentration of 10 μg ml^−1^, then fluorescence was measured by flow cytometry. The non-adherent MEC-1 cells were simply transferred in PBS added with PI to a final concentration of 10 μg ml^−1^, then fluorescence was measured by flow cytometry. Tests were performed using an Attune NxT flow cytometer (Thermo Fisher Scientific, USA) equipped with a single 488 nm laser, collecting 10 000 events per sample.

### 2.8. Multiparametric flow cytometry

The simultaneous membrane integrity and mitochondrial activity of MEC-1, A549 and HaCaT cells and HUVECs were assessed by flow cytometry after a short treatment with the peptide. For each cell line, 2.4 × 10^6^ cells were harvested as reported above (§2.3), resuspended in 1.2 ml of the corresponding medium and incubated with 24 μl of 10 μM 3,3′-dihexylocarbocyanine iodide (DiOC6) probe (FluoProbes, Interchin, Montlucon Cedex, France) at 37°C, 5% CO_2_ for 15 min. At the end of incubation, the cells were washed with PBS to remove the excess DiOC6 and resuspended in a fresh culture medium. Then PI was added at a final concentration of 10 μg ml^−1^. Finally, the peptide was added to the samples at different concentrations and incubated for 15, 30 or 60 min. Then, each sample was analysed with a flow cytometer (see above). As a positive control for loss of mitochondrial function, a cell sample was treated with a final concentration of 625 μM carbonyl cyanide-3-chlorophenylhydrazone (CCCP). Variations, within the cell population, of the mean fluorescence intensity (MFI), morphology and percentage of PI-positive cells were analysed after 15, 30 and 60 min in comparison with an untreated control.

### 2.9. *In vitro* translation

The effect of B7-005 on eukaryotic translation was determined using both a Rabbit Reticulocyte Lysate System (Promega) and a 1-Step Human Coupled IVT Kit (Thermo Scientific). The Rabbit Reticulocyte Lysate System assay was performed similarly as previously reported [25]. Briefly, 6 µl reactions, with or without B7-005 were mixed according to the manufacturer’s description and incubated for 30 min at 32°C with shaking (600 r.p.m.). Each reaction was stopped by adding 3 µl of cycloheximide (600 µM). All samples were diluted with 40 µl of luciferase assays substrate (Promega) into a white 96-well chimney flat bottom microtitre plate (Greiner). The luminescence was then measured using a Tecan Infinite M1000 plate reader. Relative values were determined by defining the luminescence value of the sample without inhibitor as 100%.

The 1-Step Human Coupled System assay was performed as follows: 6 µl reactions, with or without B7-005 were mixed according to the manufacturer’s description and incubated for 6 h at 30°C with shaking (600 r.p.m.). Each reaction was stopped by adding 3 µl of cycloheximide (600 µM). All samples were transferred into a white 96-well chimney flat bottom microtitre plate (Greiner). The fluorescence from Green Fluorescence Protein (GFP) was then measured using a Tecan Infinite M1000 plate reader. Relative values were determined by defining the fluorescence value of the sample without inhibitor as 100%. In both assays, Bac7(1–16) was also tested for the same scale as a reference.

### 2.10. *In vivo* experiments on zebrafish embryos

All experimental procedures were performed in compliance with relevant laws and institutional guidelines and were performed under the Italian Ministerial Approval (code 1FF80.N.A5X). Zebrafish eggs were placed in E3 Medium (5 mM NaCl, 0.17 mM KCl, 0.33 mM CaCl_2_, 0.33 mM MgSO_4_) and incubated at 28°C. Twenty-four hours post-fertilization (hpf), the eggs were manually dechorionated. Later, embryos were placed in E3 Medium supplemented with a final concentration of 0.2 mM phenylthiourea (PTU, Sigma-Aldrich Co.) to inhibit the production of melanin. Forty-eight hpf embryos were anaesthetized using tricaine at a final concentration of 0.02% (v/v) and placed on agarose plates, to remove the excess water and to facilitate injections. Each condition for toxicity evaluation and bacteraemia model was tested on groups of 30 embryos in at least three independent experiments. Only for biodistribution assay, groups of 10 embryos were used for two independent experiments. The injections of peptide or bacteria were performed in a volume of 4.6 nl delivered in the duct of Cuvier of embryos using capillary glass and a Nanoject II Auto-Nanoliter Injector (Drummond Scientific Co., Broomall, PA, USA) and a SteREO Microscope Discovery.V8 (Zeiss, Oberkochen, Germany, UE).

*In vivo* toxicity of B7-005 was evaluated by injecting 4.6 nl of sterile PBS containing increasing concentrations of the peptide (up to 64 mg kg^−1^) in the duct of Cuvier of embryos. PBS only was injected in untreated control groups. The toxicity of the compound was assessed evaluating the mortality of larvae, kept in E3 medium at 28°C, within 72 h after injection.

*In vivo* biodistribution of B7-005 was evaluated by injecting 4.6 nl of sterile PBS containing 1 mg kg^−1^ of fluorescent B7-005-BODIPY in the duct of Cuvier of embryos. PBS only was injected in untreated control groups. The biodistribution of the peptide was evaluated 30 min after injection using a fluorescence microscope Nikon Eclipse Ti-E live system. Meanwhile, embryos were kept in E3 Medium at 28°C. Images were analysed with Image-J software.

For the bacteraemia model, 30 ml of a mid-log phase culture of *E. coli* ATCC 25922 in MHB was centrifuged (10 min, 3000*g*), the medium was discarded, and the cells were washed twice with 30 ml of sterile PBS. Bacteria were then resuspended in a small volume of sterile PBS to a final concentration of 8.7 × 10^8^ CFU ml^−1^ and 4.6 nl of this suspension were injected in the duct of Cuvier of embryos, infecting therefore every embryo with 4000 *E. coli* cells. This bacterial load was selected for inoculation after proper setting, as it was lethal for ~70% of the infected embryos already after 24 h. The bacterial load provided by single injections was checked by injecting a drop of PBS (50 µl) that was serially diluted to determine the number of colony-forming units (CFU) on MH agar plates. A bacteria-free shot of 4.6 nl PBS only was injected in untreated control groups. The survival of fish was assessed within 72 h after the injection of larvae, kept in E3 Medium at 28°C, within 72 h after injection.

The therapeutic efficacy was investigated at first by inducing bacteraemia as reported above. Then, after 90 min to allow fish recovery from the first injection as well as bacterial spreading throughout the larvae for onset of infection, infected larvae were treated with B7-005 (in sterile PBS) at 8, 16 or 24 mg kg^−1^ by injecting 4.6 nl of peptide solution in the duct of Cuvier. After the injection, the embryos were kept at 30°C, and the survival was evaluated within 72 h after infection.

### 2.11. Statistical analysis

To assess significant differences between the experimental groups, an analysis of variance (ANOVA) was conducted followed by Tukey’s post-tests for multiple comparisons. Values of *p* ≤ 0.05 were considered statistically significant.

## 3. Results

### 3.1. Selection of bacterial resistance to B7-005

**Figure 1.**
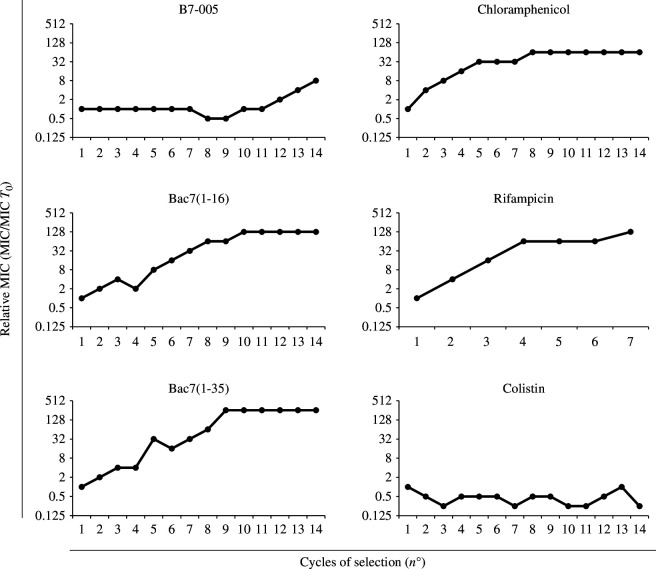
Analysis of the occurrence of bacterial-resistant mutants based on MIC. Relative MIC (MIC/MIC *T*_0_) of B7-005 and other PrAMPs and antibiotics against *E. coli* ATCC 25922 after several consecutive exposures of bacteria to sublethal amounts of antimicrobials.

It is desirable that a candidate novel antimicrobial agent exhibits delayed, or ideally little to no, emergence of bacterial resistance. It was therefore investigated whether and how rapidly *E. coli* develops resistance to B7-005. To this end, the emergence of peptide-resistant mutants was assessed by monitoring for an increase in the relative MIC of B7-005 after *E. coli* cells were repeatedly exposed to subinhibitory peptide concentrations. Bacterial cells grown in the presence of one-half times the MIC of the antimicrobial agent were subcultured and used for the following MIC assays. The relative MICs of B7-005 and the other compounds were determined against the strain *E. coli* ATCC 25922 after 14 consecutive subculturing cycles (figure 1). The absolute values of the MICs determined during the experiments are instead shown in electronic supplementary material, tables S1 and S2.

The MIC of B7-005 started to increase only after the 12th passage, whereas those of the PrAMPs Bac7(1-16) and Bac7(1-35) that were used for comparison increased after only 5 cycles. Moreover, the MIC of B7-005 was 8 μM after the 14th and final cycle, which was much lower than that of Bac7(1-16) and Bac7(1-35) that showed a MIC of 128 μM (figure 1; electronic supplementary material, table S1).

The MIC of rifampicin and chloramphenicol against *E. coli* ATCC 25922 was also determined, to compare the effect of B7-005 with that of antibiotics with intracellular targets. For both antibiotics, a 64-fold increase in MIC was observed after 4 and 8 passages, reaching 1024 μM after 7 and 8 passages, respectively (figure 1; electronic supplementary material, table S1). By contrast, the MIC of colistin, a membranolytic antibiotic with a lower propensity for bacterial resistance selection [26], remained substantially unchanged throughout the complete serial passaging process (figure 1; electronic supplementary material, table S1).

These data suggest that the emergence of resistant bacteria increases more slowly against B7-005 than against all other compounds except for colistin. This may be due to an antimicrobial mechanism of action of B7-005 that is less dependent on the bacterial transporters, such as SbmA. Moreover, the results obtained here are in agreement with similar experiments performed previously with Bac7(1-16), chloramphenicol and colistin [27].

### 3.2. Effect of human serum on the activity of B7-005

The bioavailability in human serum of a peptide intended for clinical systemic use is a critical parameter for the success of peptide-based drugs [28]. Previous studies have already excluded rapid proteolytic degradation of B7-005 by serum proteases, since its antimicrobial activity was only slightly reduced in the presence of 10% (v/v) human serum [20]. However, irreversible sequestration of the peptide by serum proteins at higher serum concentrations could also impair its antimicrobial activity. To check if the peptide maintained its availability under conditions closer to the clinical ones, the MIC of B7-005 against the *E. coli* reference strain ATCC 25922 was determined in the presence of increasing serum concentrations, up to 50% (v/v). As seen in table 1, the MIC of B7-005 doubled (from 1 µM to 2 µM) in the presence of 20–30% (v/v) serum, and further increased to 4 µM and 8 µM in the presence of 40% and 50% (v/v) serum respectively. These results indicate that serum has only a partial inhibitory effect on the antimicrobial activity of the peptide, which retains its overall antimicrobial activity.

**Table 1.** MIC of B7-005 towards *E. coli* ATCC 25922 in the presence of increasing concentrations of serum in Müller–Hinton broth.

	MIC^a^ of B7-005					
	human serum (% v/v) in MH medium					
	0%	20%	25%	30%	40%	50%
*E. coli* ATCC 25922	1 µM	2 µM	2 µM	2 µM	4 µM	8 µM

^a^ MIC calculated by visual inspection of the plate, as the first clear well after 18 h at 37°C. Results are the mode of three independent experiments (*n* = 3).

### 3.3. Effects of B7-005 on human cells

**Figure 2.**
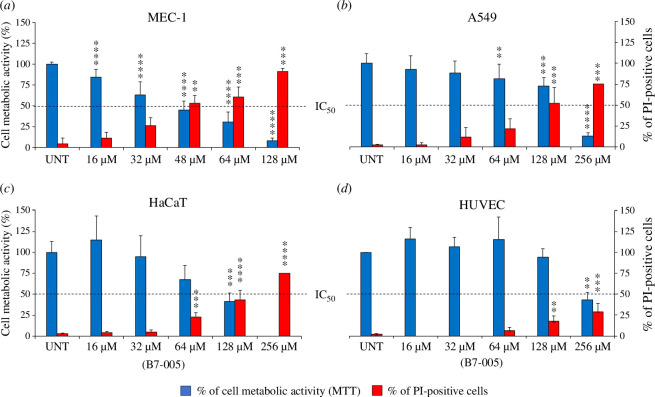
Effect of B7-005 on human cells. MEC-1 (*a*), A549 (*b*), HaCaT (*c*) and HUVEC (*d*) were exposed to the peptide for 24 h. Cell viability (left *y*-scale) and membrane integrity (right *y*-scale) were determined by measuring the metabolic activity by MTT/S assay (blue bars) and the PI uptake (red bars), respectively. Untreated samples (UNT) received water instead of peptide. Average and standard error of the mean of 3 independent experiments (PI, *n* = 3) or average and standard deviation of 3 independent experiments performed as technical triplicate (MTT, *n* ≥ 9). *p ≤ 0.05, **p ≤ 0.005, ***p ≤ 0.0005, ****p < 0.0001 treated versus untreated cells (UNT) (Tukey’s test and ordinary one-way ANOVA).

To investigate the potential cytotoxic effect of B7-005 on human cells and tissues, human cancer cells (MEC-1, lymphocyte precursors; and A549, adenocarcinomatous alveolar basal cells), immortalized cells (HaCaT, epidermal keratinocytes) and primary healthy cells (HUVECs) were exposed to increasing concentrations of the peptide. Following treatment, the viability of the cells was assessed by monitoring their metabolic activity with the MTTs colorimetric assay (blue bars in figure 2*a–d*), as well as by checking the integrity of their membranes by measuring the cellular uptake of PI (red bars in figure 2*a–d*). B7-005 induced a concentration-dependent loss of MEC-1 cell viability after 24 h of incubation. According to MTT assays, the peptide displayed an IC_50_ value close to 48 μM—a concentration that also permeabilized ~50% of the cells (figure 2*a*). The IC_50_ value of B7-005 for A549 cell viability was between 128 μM and 256 μM and slightly below 128 μM for HaCaT cells. Again, the decrease in metabolic activity paralleled the degree of membrane permeabilization, as in the presence of 128 μM B7-005, ~50% of the cell population of both cell lines were permeabilized (figure 2*b,c*). Lastly, the IC_50_ for HUVECs was ~256 μM, a value that was even higher than for the other cell types. At 256 μM, B7-005 permeabilized slightly less than 30% of the cell population (figure 2*d*). Overall, all cell types exhibited low susceptibility to the peptide, albeit to varying degrees. MEC-1 cells were the most sensitive to B7-005. Interestingly, primary HUVECs were the least susceptible cells showing no detectable effects up to a concentration of 128 µM, which was more than 100-fold higher than the inhibitory (electronic supplementary material, table S1) and microbicidal [20] concentration observed for B7-005 towards *E. coli* ATCC 25922. The susceptibility of HaCaT and A549 cells to B7-005 was in between, with the latter cell line being less sensitive to the peptide than the former. Interestingly, the IC_50_ values for all cells were well above the MIC of B7-005 towards *E. coli* in the presence of high serum concentrations (table 1).

### 3.4. Effect of short exposure of human cells to toxic concentrations of B7-005

**Figure 3.**
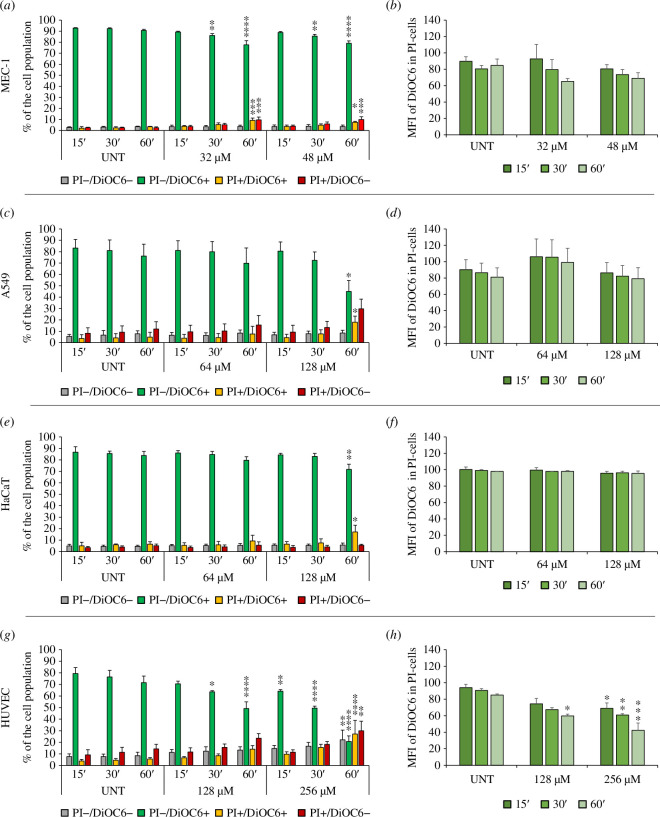
Effects of acute exposure to high concentrations of B7-005 on MEC-1, A549, HaCaT and HUVEC cells evaluated by flow cytometry. Untreated controls (UNTs) received water instead of B7-005. (*a,c,e,g)* Data indicate the percentages of cells stained with DiOC6, PI or both. Average and standard deviation of three independent experiments are shown. (*b,d,f,h) *The fluorescence intensity of DiOC6 stained cells of PI-negative cells and expressed as a percentage compared with UNTs (100%) at timepoint 0 min. Average and standard error of the mean of three independent experiments are shown. *p ≤ 0.05, **p ≤ 0.005, ***p ≤ 0.0005, ****p < 0.0001; treatment versus UNT at the same timepoint, Tukey’s test and two-way ANOVA.

To date, only a few hints on the mode of action of B7-005 were available, limited to *E. coli*. No information was available for eukaryotic cells. Unfortunately, the treatment of cells with the peptide for 24 h did not distinguish whether the cell permeabilization was the main cause of cell death or rather a consequence of cell death arising during the incubation. To address this, cells were exposed to B7-005 for shorter periods of time (15–60 min), and a bi-parametric flow cytometric analysis was performed (figure 3). For each condition, the integrity of the cell membrane (propidium iodide positive cells, PI+) as well as the polarization of the mitochondrial membrane (DiOC6 positive cells, DiOC6+) were examined. Data from PI uptake and positivity for DiOC6 were combined to profile and categorize cells as follows. PI−/DiOC6+ indicated intact and metabolically active cells. PI+/DiOC6+ indicated membrane-damaged cells that still have functional mitochondria. PI+/DiOC6− displayed permeabilized plasmatic membrane with depolarized mitochondrial membrane and, finally, PI−/DiOC6− indicated non-permeabilized cells showing depolarized mitochondria. Each cell type underwent incubation with B7-005 for 15, 30 or 60 min at two concentrations approximating the respective IC_50_ calculated at 24 h (refer to figure 2). Then, the percentages of the cell population positive or negative for the two dyes were recorded (see figure 3). A 60-min treatment with B7-005 had a significant, but mild impact on the membrane integrity or mitochondrial activity of the MEC-1 cells, since only a 20% reduction of the PI−/DiOC6+ cells was observed (figure 3*a,b*). Conversely, in A549 cells, a more notable effect was observed, with ~50% of the cell population losing membrane integrity (PI+) after 60 min of treatment at 128 µM (≈IC_50_). Among permeabilized cells, around 20% remained positive for DiOC6 staining (PI+/DiOC6+), indicating residual activity of mitochondria, while 30% also lost mitochondrial functionality (PI+/DiOC6−) (figure 3*c*). However, intact A549 cells, negative to PI, did not exhibit a substantial quantitative drop in the polarization of the mitochondrial membrane (PI−/DiOC6+), suggesting that in the absence of cell membrane damage, there was no specific intracellular effect of B7-005 on mitochondria (figure 3*d*).

Exposing HaCaT cells to B7-005 under the same conditions (60 min, 128 µM), ~25% of the cell population was permeabilized, and, unlike A549, most of the permeabilized HaCaT cells maintained polarized mitochondria (PI+/DiOC6+, ≈15%). The fraction of cells losing mitochondrial activity was minimal, around 5% (figure 3*e*). Moreover, B7-005 did not significantly affect the polarization of the mitochondrial membrane in intact cells, suggesting no specific targeting to this organelle (figure 3*f*).

The treatment of HUVECs with the peptide had the strongest effect among the tested cell types. After 60 min of exposure to 256 µM B7-005, only about 20% of cells remained intact with polarized mitochondria (PI−/DiOC6+). Most cells lost membrane integrity (PI+, ≈60%), and approximately one-half of these cells (≈30% of the total) had no signs of mitochondrial activity (PI+/DiOC6−). Notably, HUVECs were the only cell type where 20% of the cell population lost mitochondrial membrane polarization in the absence of cell membrane permeabilization (PI−/DiOC6−) (figure 3*g*). Consistent with this observation, there was a significant drop in the fluorescence intensity of DiOC6 in cells with intact membranes, suggesting a direct activity of B7-005 on these organelles (figure 3*h*).

Overall, cytotoxic concentrations of B7-005 exerted variable effects on cells according to the cell type under study, differing in the extent of cell membrane permeabilization and for the targeted cellular structure. The effects of B7-005 ranged from mainly membrane-destabilizing activity to a mixed mode of action involving also non-lytic toxic events targeting mitochondria, suggesting a complex interaction of the peptide with the cell. Nevertheless, generally, cell damage occurred only in the presence of the highest concentration of B7-005 and after a long exposure, stressing again the tolerability of the peptide by human cells.

### 3.5. Effect of B7-005 on eukaryotic protein synthesis

**Figure 4.**
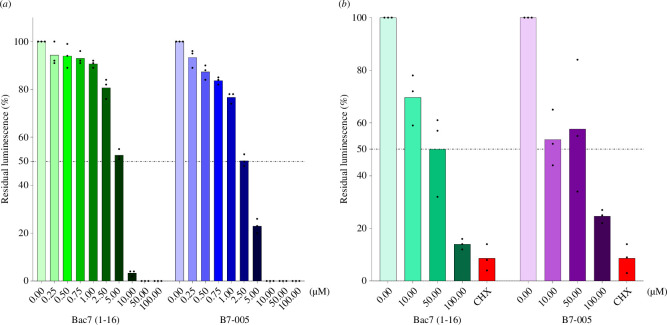
Effect of increasing concentrations of B7-005 and Bac7(1-16) on *in vitro* translation reactions. A rabbit reticulocyte-based system expressing firefly luciferase (*a*) or a human HeLa cell-based system expressing GFP (*b*) were used. Histograms represent the mean of independent triplicates, whose single results are reported as black dots. The luminescence and the fluorescence, respectively, were normalized on that measured in the absence of peptide, which was assigned as 100%.

B7-005 is a potent inhibitor of bacterial protein synthesis, as demonstrated using *E. coli* lysates [20]. To investigate whether this peptide can also affect eukaryotic protein synthesis, *in vitro* translation assays were performed by using two commercial systems, namely (i) rabbit reticulocyte lysates [25] and (ii) lysates of human cell line HeLa, expressing a luciferase or GFP reporter, respectively (figure 4). A reduction in the production of the reporters in the presence of the peptide would suggest an inhibitory effect towards eukaryotic protein synthesis. In parallel, Bac7(1-16) was used as a positive control, since its effect on eukaryotic translation has already been reported with an IC_50_ of 2.5 µM [25]. Both B7-005 and Bac7(1-16) inhibited protein synthesis in the rabbit reticulocyte system, displaying comparable potency with IC_50_ values of 2.5 µM and 5 µM, respectively (figure 4*a*). Results obtained with Bac7(1-16) were consistent with previous studies using this peptide, where an IC_50_ of 2.5 µM was reported [25]. The inhibition of protein synthesis by B7-005 in the reticulocyte system (figure 4*a*) was more pronounced than in HeLa cell lysates (figure 4*b*). B7-005, like Bac7(1–16), is a potent inhibitor of eukaryotic translation machinery, although with slightly minor efficacy with respect to prokaryotic protein synthesis. In fact, B7-005 and Bac7(1-16) inhibited the bacterial translation in an *E. coli* system with IC_50_ values of 1 µM [20] and 0.75–1 µM [20,25], respectively. This makes B7-005 selective for bacterial protein synthesis, but less specific than Bac7(1-16) for this prokaryotic biosynthetic pathway.

### 3.6. B7-005 promotes the survival of zebrafish embryos in a model of *E. coli* bacteraemia

**Figure 5.**
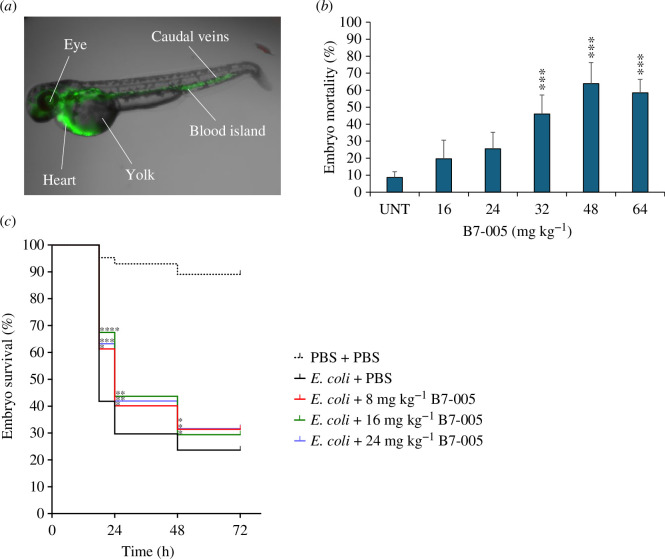
*In vivo* evaluation of B7-005 effects on zebrafish embryos. (*a*) Biodistribution of B7-005-BY through zebrafish larvae was assessed 30 min after injection by fluorescence microscopy. (*b*) Systemic toxicity of B7-005 was evaluated as the percentage of mortality of larvae injected with increasing concentrations of the peptide or with PBS only (UNT) within 72 h after injection. The error bars represent the standard error of the mean calculated from at least three independent experiments. One-way ANOVA, UNT versus treated samples. (*c*) Survival rate of zebrafish embryos, infected with a lethal dose of *E. coli* ATCC 25922 and subsequently treated with B7-005 90 min post-infection. Control groups received only PBS instead of the peptide (*E. coli* + PBS) or instead of both the peptide and the bacteria (PBS + PBS). Log-rank Mantel–Cox test.

Since eukaryotic cells, and particularly normal endothelial cells, were not strongly affected by 24 h exposures to bactericidal concentrations of B7-005, we investigated the *in vivo* efficacy of B7-005 to counteract bloodstream infections using a zebrafish embryo model system. The biodistribution of the B7-005 in the embryos, its acute systemic toxicity and its *in vivo* antibacterial activity in a model of systemic bacteraemia were assessed. First, we investigated whether intravenous administration of B7-005 allowed the peptide to evenly distribute through the vascular system of the fish larvae. A fluorescent derivative of B7-005 labelled with BODIPY-FL (B7-005-BY) was injected at 1 mg kg^−1^ into the bloodstream of the embryos. Fluorescence microscopy confirmed that the compound was evenly distributed throughout the vascular system within 30 min post-injection (figure 5*a*).

To investigate the possible toxicity of B7-005 to fish larvae, increasing amounts of B7-005 were injected into the bloodstream of different larvae cohorts. The survival rate of the fish embryos was then recorded for 72 h post-treatment. B7-005 began to significantly reduce the viability of the larvae at a concentration of 32 mg kg^−1^ where it was lethal for ~40% of the larvae within 72 h (figure 5*b*). A threshold concentration of 24 mg kg^−1^ was therefore chosen for the subsequent experiments, as mortality did not exceed 20%, which is close to the mortality rate due to the injection procedure only, as observed in the untreated control group (10%).

To assess the *in vivo* efficacy of B7-005, a model of lethal *E. coli* bacteraemia was set up using zebrafish embryos with modifications compared to previous studies [29,30]. A bacterial load of 4000 *E. coli* ATCC 25922 cells per fish was chosen for subsequent experiments as 70% of the animals died within 24 h after infection (electronic supplementary material, figure S1). It was therefore possible to set a lethal systemic infection using *E. coli* ATCC 25922. To test the efficacy of B7-005 *in vivo*, zebrafish larvae were infected as above, and, after waiting 90 min for the onset of infection, the B7-005 peptide was intravenously injected into the larvae at three different concentrations (8, 16 and 24 mg kg^−1^). B7-005 significantly improved the survival rate of the embryos with respect to the control group of larvae treated with PBS at all tested concentrations. The best result was obtained using 16 mg kg^−1^ B7-005, which nearly doubled the number of survivors 18 h post-infection. The effect of the peptide was maximal at this time point. In fact, the difference in the number of survivors between treated and untreated groups of larvae decreased over time, still remaining significant, within the first 48 h, but then remained unchanged until the end of the experiment, 72 h after infection (figure 5*c*). B7-005 thus maintained its antimicrobial activity also *in vivo* and improved the survival rate of zebrafish embryos affected by a systemic infection of *E. coli*.

## 4. Discussion

The need for new antimicrobial compounds is stimulating the research on antimicrobial peptides. However, to date, despite numerous studies on AMPs, only very few of these molecules have entered into the clinical pipeline [31]. The reason for this high failure rate has usually been (i) the poor stability of AMPs, (ii) their high cytotoxicity, and (iii) their modest antimicrobial efficacy once tested *in vivo* [28,31]. Moreover, in the age of antimicrobial resistance, another parameter has also gained prominent importance, namely, that any antimicrobial compound intended to become a new antibiotic must undergo rigorous evaluation for its ability to avoid the emergence of resistance mechanisms in bacteria.

In our study, we demonstrate that B7-005 displayed a notable advantage over the related PrAMPs Bac7(1-16) and Bac7(1-35) in preventing the development of resistance during experiments designed to select resistant strains (figure 1). This feature may stem from the mode of action of B7-005, encompassing the mild destabilization of bacterial membranes, which likely facilitates self-promoted uptake phenomena. Such a mechanism would make B7-005 less dependent on transport via the bacterial inner membrane transporter SbmA compared to other native PrAMPs [20]. In this manner, any loss of SbmA by bacteria would make microorganisms less sensitive to Bac7 peptides but not to the B7-005 peptide. Furthermore, B7-005 endured for longer before bacteria developed resistance when compared to two out of the other three tested antibiotics, including chloramphenicol, which, like B7-005, targets protein synthesis [32]. The only compound that surpassed B7-005 in preventing the emergence of bacterial resistance was colistin, a lipopeptide antibiotic targeting a very common and accessible bacterial structure, such as the lipopolysaccharides on the membranes of Gram-negative bacteria [33]. Interestingly, in the past, a modified version of Bac7(1-16) bearing a C-terminal 12-mer carbon chain (Bac7-C12) performed similar to colistin when tested for the emergence of bacterial resistance. However, Bac7-C12 displayed marked membranolytic activity, so cannot be compared with ordinary PrAMPs that specifically target protein synthesis [27]. Collectively, this suggests that B7-005 emerges as a PrAMP whose antimicrobial mode of action cannot be easily evaded by bacteria.

The resistance of antimicrobial peptides to proteases represents another problematic issue. However, when the stability and efficacy of B7-005 were tested in a context closer to the clinical one (i.e. up to 50% v/v human serum), B7-005 retained its antimicrobial activity. In fact, B7-005 maintained a MIC of 8 µM towards *E. coli* (see table 1), supporting previous results on the stability of B7-005 towards proteolysis *ex vivo* [20] and suggesting that it does not undergo irreversible inactivation via sequestration by serum components. The interaction of B7-005 with serum components may be further explored in the future, and may even be exploited for tuning its bioavailability as has been done for other peptide drugs [21].

We expanded the cytotoxicity evaluation of B7-005 towards multiple cell types derived from different body districts. Microbicidal concentrations of B7-005 were shown to be safe for human cells of various nature. The presence of bulky hydrophobic residues in the PrAMPs sequence often increased the affinity of these peptides for biological membranes and this has been associated with an enhanced risk of cytotoxicity [17,18]. However, this was not the case with B7-005. The IC_50_ values of the peptide calculated for each cell line were considerably higher than the MIC values of the compound towards *E. coli*, even in the presence of human serum (table 1, figure 1) and towards other clinically relevant pathogens tested previously [20].

In this study, the response of cells to B7-005 varied according to the cell type. The tumoral MEC-1 cells were the most sensitive to the cytotoxic effects of the peptide, displaying even higher sensitivity to B7-005 than observed in a previous study [20]. However, we note that in the previous study, the peptides were generated by SPOT synthesis and therefore were of lower-grade purification, which likely explains the differences observed with our study. Conversely, it is worth noting that HUVECs exhibited the least susceptibility to B7-005. These cells were the only non-immortalized or tumoral cells and their limited susceptibility may arise precisely from their non-tumoral nature. Tumour cell lines often have anionic phospholipids on their outer membrane leaflet, rendering them more electrostatically attractive for cationic AMPs, and thus more exposed to their cytotoxic effects [34]. However, the observation that tumoral cells, such as those of the osteosarcoma cell line MG-63, remained unaffected by B7-005 up to 128 µg ml^−1^ (equivalent to 54 µM) [35] suggests that factors other than outer membrane phospholipid composition need to be considered to explain the variation in cytotoxicity. The diversity in sensitivity across various cell types emphasizes the importance of tailoring the use of optimized AMPs according to the final application, in order to find the body districts where these peptides may find their most efficient and safe application.

Exposure of cells to B7-005 for 24 h was not sufficient to distinguish if membrane permeabilization was the main cause or a consequence of the observed cytotoxicity. B7-005 may have entered eukaryotic cells without membrane damage and then targeted cytosolic structures, including but not limited to the translational machinery. To investigate this aspect, cells were challenged with short exposure to high concentrations of B7-005. The evaluation of membrane integrity was no more flanked by the assessment of metabolic activity based on the general functionality of mitochondria (e.g. formazan salts assay), but rather by a more specific evaluation of mitochondrial membrane depolarization (DiOC6−). In fact, it would not have been the first time that an AMP targets mitochondria [36]. Confirmation of the hypothesis that B7-005 targets mitochondria was limited to the HUVEC model. In fact, in the presence of B7-005, only HUVECs displayed a significant concomitant reduction in the population of intact viable cells (PI−/DiOC6+) and in the fluorescence intensity of DiOC6 in non-permeabilized cells (PI−), indicating mitochondrial depolarization (figure 3*h*). Data suggested that B7-005 had a direct impact on the mitochondrial potential, which was not an indirect effect of the permeabilization of the cellular membrane, and that hints at mitochondrial dysfunction. Conversely, in other cell types such as A549 and HaCaT, the decrease of the number of intact viable and metabolically active cells (PI−/DiOC6+) occurred concurrently with membrane permeabilization (PI+ cells), notably after 60 min in the presence of 128 µM B7-005 (figure 3*c,e*). This suggests that the cytotoxic mechanism of B7-005 on A549 and HaCat cells involves primarily the initial membrane damage, potentially followed by secondary mitochondrial depolarization and consequent dysfunction. The mechanism of cytotoxicity of B7-005 on MEC-1 cells, which exhibited the lowest tolerance to the peptide, remained unclear. In fact, although exposure to B7-005 for 24 h led to significant membrane damage (figure 2*a*), short-term exposure (1 h) resulted in minimal membrane permeabilization (figure 3*a*). Additionally, unlike HUVECs, there was no evidence of mitochondrial depolarization in MEC-1 cells. Thus, the data did not indicate whether the primary cytotoxic mechanism was cell membrane permeabilization or mitochondrial damage. In each case, the cytotoxic effects of B7-005 on MEC-1 were slower than that on the other cell lines since they were not detected in the experimental timeframe. Overall, the results of short exposure of cells to B7-005 again underlined differences in response to the peptide. This may be due to dissimilarities in the mode of action of the compound according to the different physiology of the tested cell types. The use in this work of a panel of human cells of diverse origins demonstrated how complex, different and polyhedric can be the interaction of PrAMPs with eukaryotic cells. We shed light on some aspects of the interaction of PrAMPs with human cells, showing a warp which is even more complex than expected, and that will be a fascinating field of investigation to be addressed at various levels of analysis.

A correlation between the reduction in mitochondrial functionality and the inhibition of translation cannot be ruled out. Protein synthesis in eukaryotic cells was inhibited *in vitro* by B7-005 at slightly higher concentrations than those required to inhibit prokaryotic synthesis. Indeed, the peptide displayed an IC_50_ of 2.5 µM and 10 µM for the rabbit and human *in vitro* translation systems, respectively, compared with the previously calculated IC_50_ = 1 µM for the *E. coli in vitro* translation system [20]. Therefore, this compound is only partially selective for bacterial translation and could potentially target also the mitochondrial ribosomes. Due to their bacterial origin, mitochondria are sensitive to several inhibitors that target prokaryotic protein synthesis [37]. Thus, it can be hypothesized that mitochondria may represent a secondary target for B7-005 at high concentrations, also in the absence of cell membrane damage, likely through the inhibition of eukaryotic mitochondrial protein synthesis.

Then, coming back from the mode of action to the generic evaluation of toxicity, the high tolerability of B7-005 observed *in vitro* by cells was also confirmed *in vivo*. The experiments on zebrafish larvae reported that B7-005 was safely and efficaciously applied to treat a severe infection, like bacteraemia (figure 5). The zebrafish embryos in fact tolerated a high amount of the peptide, up to 24 mg kg^−1^, and the treatment of infected larvae with 16 mg kg^−1^ B7-005 halved the lethality of the *E. coli* systemic infection.

In the current work, the infection of zebrafish larvae with *E. coli* and their treatment with B7-005 were strictly controlled performing them by microinjection in the Cuvier duct [29,30]. The model we established using *E. coli* ATCC 25922 demonstrated a substantial mortality rate (~70% within 24 h). While *E. coli* ATCC 25922 did not succeed in causing mortality across the entire larval cohort as reported, for example, for the *E. coli* strain K46 [38], it is noteworthy that, conversely, additional strains of *E. coli* failed to induce lethal infections under similar conditions [38]. To better simulate the clinical treatment of infectious disease, antimicrobial treatments have been administered post-infection, rather than given simultaneously with bacteria [39]. Despite these challenging conditions, B7-005 was more effective than Bac5 peptide, another PrAMP previously used in a zebrafish larvae infection model [40]. In fact, Bac5 was only effective when the pathogens and peptide were simultaneously injected into embryos, but the benefit of the treatment disappeared when the administration of the compound occurred post-infection [40]. Although the model of zebrafish larvae has become attractive to perform *in vivo* evaluation of antimicrobial activity for antibiotic compounds [30], it is difficult to compare different studies performed on antimicrobials on zebrafish embryos due to differences in pathogens, infection models, administration route and timing of the treatment. For example, some studies have been performed adding antimicrobials, pathogens, or both, into water [41], but this is poorly representative of the treatment of a human bloodstream infection. The optimal timings, infection route and bacterial load identified for the infection model were quite in line with previous experimentation performed with quite similar experimental designs but using another Gram-negative pathogen [42]. The B7-005 distributed homogeneously in the bloodstream of fish larvae (figure 5*a*). This is not always the case since harmful or lethal macroscopic aggregation of the amphipathic B7-005 could have occurred *in vivo* in the bloodstream, which would have been difficult to predict on the basis of the experiments performed in the presence of human serum (table 1).

The most evident effect of B7-005 in increasing the survival of larvae was reported after 18 h. Subsequently, the advantage between treated and untreated animals decreases over time, still remaining statistically significant for the whole duration of the experiment. The promising result of almost doubling the surviving larvae with respect to the untreated cohort by treating infected embryos with 16 mg kg^−1^ of B7-005 may have been even improved by repeated administrations of the peptide. Unfortunately, the selected protocol excluded the possibility of further administrations of the compound during the treatment. Additional injections may have been too stressful for fish larvae since they had already received two shots (infection and peptide). In the future, new technical developments in microinjections may make it possible to increase the number of subsequent administrations, unlocking the possibility of better exploring the *in vivo* potential of B7-005 in zebrafish larvae.

In summary, B7-005 demonstrates both excellent tolerance and effectiveness *in vitro* and *in vivo*, and its minimal propensity to promote antimicrobial resistance suggests its clinical utility could be long-lasting. B7-005 is a promising candidate for the development of next-generation antibiotics. Other designer PrAMPs, A3-APO, Api88 and Onc72, tested in murine models of bacteraemia, alone or in combination with antibiotics, were helpful in promoting the survival of animals [43–46]. Therefore, we strongly believe that also B7-005 could help in curbing the rise of antibiotic-resistant pathogens.

## Data Availability

We do not have raw data like genomic or proteomic data. Most of our data are numeric whose entity may be derived directly by the figures published in the paper or by visualization of a fluorescence microscopy picture that did not require data refining. The only exception is figure 1, which reports data as relative MIC and no indication is available in the text of the absolute values of the MIC to understand the potency of the compound *per se*. To fix this, these data have been made available in the electronic supplementary material [47].
